# An Open Letter

**Published:** 1893-10

**Authors:** 


### Editorial.

### AN OPEN LETTER.

In an open letter in this issue we desire to call attention to a plagairistic article published in the Transactions of the Georgia Medical Association for this year.

The claims made in this letter seem to be only too well established, and it is a reflection both upon the Association and its Transactions, that the latter should go out containing an article of this kind.

A paper, when published in the Transactions, apparently comes with it the approval of the Association, or at least creates that impression upon the profession, and in this way gives weight to any article published in it. For any article in the Transactions, or republished from it, is usually supposed to have been read before a body of intelligent medical men. In regard to the paper mentioned this was not true, as the essay was read only by title.

The laws of the Association allows any member who puts the title of a paper on the programme, to publish his paper in the Transactions without reading it at the meeting.

Now we consider this a very unwise policy, and so long as it is persisted in, there will appear in the Transactions papers that are unworthy of a place in these volumes, and their authors would never have read before the members. There are several papers of this character in the present volume.

It is true there is a Committee on Publications, whose duty it is to decide what papers should appear in these volumes. But no matter how anxious the members of this committee may be to perform their duties, the volume of work is so great that it is impossible for them to do justice to it.

There were forty-eight papers on the last programme, only twenty of which were read before the Association, leaving twenty-eight papers, the contents of which the Association knew nothing; privileged to appear in their Transactions, nor was the Publication Committee much wiser.

Over one-third of the reading matter of the present volume, I know had never been seen by any one except the author of the paper and the proof reader when the volume was sent out.

We think it would be a wise policy for the Association to pass a rule not allowing any paper to appear in the Transactions but those read at the meeting, and if this is done we feel certain that the character of the papers will be materially improved. D. H. H.

